# Scales assessing L2 speaking anxiety: Development, validation, and application

**DOI:** 10.3389/fpsyg.2022.972671

**Published:** 2022-10-20

**Authors:** Jie Gao

**Affiliations:** College of Foreign Languages and Literature, Fudan University, Shanghai, China

**Keywords:** anxiety assessment scales, L2 speaking anxiety, quantitative research methods, scale development, scale validity

## Abstract

Through featuring a historical review of the L2 speaking assessment scales applied in related studies, this paper targets at providing responses for the following three questions (a) How are the scales assessing L2 speaking anxiety developed and adapted in related research? (b) What are the frequently adopted methods for validating speaking anxiety scales? (c) How is L2 speaking anxiety represented and interpreted with a dynamic approach? Based on analyzing the development process of frequently-used scales for assessing test anxiety, foreign language classroom anxiety, and speaking anxiety, the author classified the scales into three categories: test-based scales measuring speaking anxiety, classroom-based scales measuring speaking anxiety, and activity-based scales measuring L2 speaking anxiety. As for the scale validation methods, Classical Testing Theory (CTT) and Rasch measurement were introduced as two major statistical paradigms for guaranteeing the reliability of the scales. This paper also summarizes the emerging themes generalized from research focusing speaking anxiety assessment, where the dynamic approach is discussed as a guideline to interpret the relationship among anxiety, language performance, and other factors involved in language learning. This paper ends with highlighting possible directions for anxiety-related research in the future, where technology intervention and the “positivity ratio” might become new attempts for pedagogical design.

## Introduction

During the language learning process of second language speakers, the relationship between anxiety and their language performance has often been considered as a negative one. As a subjective feeling filled with “tension, apprehension, nervousness, and worry associated with an arousal of the autonomic nervous system” (Spielberger et al., 1983, p. 1), anxiety has been identified as the reason for causing unsatisfying language performance (Zhang, 2019), reducing language learners' willingness to communicate (Liu, 2018; Jiang and Dewaele, 2019), debilitating speakers' abilities in demonstrating critical thinking (Blume et al., 2010), and projecting a personal image that lacks communicative confidence (Araki and Raphael, 2018; Mulyono and Saskia, 2021). Language teachers and learners are endeavoring to search for coping mechanisms to tackle anxiety, which has led to fruitful research outcome in identifying the sources of anxiety, as well as explicating the relationship between anxiety and other factors involved in language learning.

The measurement of speaking anxiety, or the transformation of speaking anxiety to a quantitative variable, heavily relies on the use of assessment scales. An accurate estimation of the anxiety perceived by L2 language learners not only presents solid data for further statistical analysis, but also reflects researchers' understanding of anxiety as an affective variable. This paper presents a narrative review of the scales used for assessing L2 speaking anxiety, and responds to the following research questions:

(a) How are the scales assessing L2 speaking anxiety developed and adapted in related research?
(b) What are the frequently adopted methods for examining the validity and reliability of speaking anxiety scales?
(c) How is L2 speaking anxiety represented and interpreted with a dynamic approach?

The development of scales assessing speaking anxiety was initiated with an explanation of anxiety as a general concept. Commonly-used frameworks have categorized anxiety as trait anxiety and state anxiety (Spielberger, 1966; Scovel, 1978), or facilitating anxiety and debilitating anxiety (Alpert and Haber, 1960). While trait anxiety remains constant across different contexts, state anxiety varies with changes that occur to specific circumstances. Foreign Language Anxiety (FLA), which encompasses L2 speaking anxiety, is considered as a state-related anxiety that is situation specific. This “situation specific” property of FLA, which is attributed to its persistency and multi-facetness (MacIntyre and Gardner, 1991; MacIntyre, 1999, 2007; Horwitz, 2010) has resulted in its frequent juxtaposition with test anxiety. It is highly possible that the evaluation of learners' language performance takes place in a testing environment. As was described by Pintrich and Schunk (2014, p. 265), test anxiety refers to “a set of phenomenological, physiological, and behavioral responses” caused by the fear of negative outcome or failure in evaluative situations such as examinations. The division between facilitating and debilitating anxiety, however, is dangerous and problematic according to Horwitz (2017). Placing facilitating anxiety and debilitating anxiety on the opposite end is eliminating the possible relatedness and interaction between the two, which might lead to a complete denial of the potential “positiveness” within certain types of anxieties. This interpretation of anxiety, or the confirmation of anxiety's multi-facetness, resonates with MacIntyre's (2017, p. 16) explanation for a dynamic approach to understanding anxiety:

“This new, emerging tradition emphasizes situating anxiety among the multitude of interacting factors that affect language learning and development. Anxiety is continuously interacting with a number of other learner, situational, and other factors including linguistic abilities, physiological reactions, self-related appraisals, pragmatics, interpersonal relationships, specific topics being discussed, type of setting in which people are interacting, and so on.”

Following the dynamic approach of interpreting Foreign Language Anxiety (FLA), this paper navigates the scales that are developed for measuring L2 speaking anxiety, which is closely related to test anxiety and classroom learning anxiety. This paper also analyzes the methods for examining the validity and reliability of scales measuring L2 speaking anxiety, and identifies themes emerging from research that applies L2 speaking anxiety scales. In the last section of this paper, suggestions are provided for the design and adoption of scales in the future, when language learning and communication are hugely intervened by online instructional methods and technologies in diverse forms.

## Methods of article review

In response to the first question that examines the development and adaptation of L2 speaking anxiety assessment scales, the author adopted a historical review approach and started with investigating the scales that measure anxiety as a general concept. L2 speaking anxiety, which occurs in language assessment situations as well as daily communication scenarios, has been measured both in a testing environment and language classrooms. For this reason, the focus points of investigation also locate on scales in measurement of testing anxiety and language learning anxiety in classrooms. Learners, however, often experience speaking anxiety when participating in specific activities, because L2 speaking has also been concretized by various pedagogical practices in language classrooms. The synthesis of scales for assessing L2 speaking anxiety thus follows the outline of examining test-based scales measuring anxiety, classroom-based scales measuring anxiety, and activity-based scales measuring anxiety. The literature cited, which represents the first group of studies in discussion of relevant assessment scales, provides important content materials for scale revision and adaptation in a broader range of research related to speaking anxiety.

To provide answers for the second and third research question, the author combed through the most recent research with the keywords of “foreign language anxiety,” “L2 speaking anxiety,” “assessment scales,” and “scale reliability and validity”. The articles collected by the author have fulfilled the following requirements:

(1) L2 speaking anxiety is evaluated by scales as an individual dimension or a component embedded in FLA assessment.
(2) The articles have reported empirical research results regarding L2 anxiety assessment.
(3) The articles are published in peer-reviewed journals and book chapters after the year of 2010.

As is shown in Table 1, a total number of 49 articles were included in this literature review process. The author identified a list of topics from the studies based on the assessment purposes of the scales, and grouped these topics into more overarching themes that summarize the functioning of scales in speaking anxiety research. Detailed interpretation of the themes is presented in later sections of this paper, which embodies the dynamic approach in emphasis of speaking anxiety and its interaction with other factors.

**Table 1:** Articles reviewed in identification of scales assessing L2 speaking anxiety.

**Theme**	**Topic**	**No. of articles**	**Publication**
The validity and reliability of L2 speaking anxiety scales are examined through multiple statistical procedures.	L2 speaking anxiety scales development and validation	6	- Ali, 2016 - Ali, 2017 - Apple, 2013 - Park, 2014 - Taat et al., 2020 - Yaikhong and Usaha, 2012
L2 speaking anxiety scales are adopted to assess pedagogical outcome.	The effects of English-medium instruction and classroom pedagogies on FLA	8	- Chou, 2018 - Galante, 2018 - Kralova et al., 2017 - Lee, 2016 - Liu, 2021 - Liu and Xiangming, 2019 - Jin et al., 2021 - Scida and Jones, 2017
	The effects of instructional technologies on FLA	10	- Aldukhayel, 2022 - Bashori et al., 2022 - Bárkányi, 2021 - Chen and Hwang, 2020 - Chen et al., 2022 - Chen and Lee, 2011 - Jebali, 2014 - Pan et al., 2022 - Xiangming et al., 2020 - York et al., 2021
	The effects of assessment approaches on FLA	3	- Estaji and Farahanynia, 2019 - Sohrabi and Ahmadi Safa, 2020 - Zheng et al., 2021
L2 speaking anxiety scales are adopted to unpack the relationship between anxiety and affective variables.	Identification of factors contributing to anxiety	3	- Mak, 2011 - Öztürk and Gürbüz, 2013 - Sun and Teng, 2021
	Interaction between FLA and Foreign Language Enjoyment (FLE)	4	- Chen et al., 2021 - Dewaele and Alfawzan, 2018 - Dewaele and MacIntyre, 2014 - Jiang and Dewaele, 2019
	Relationship between FLA and other affective variables (e.g., Willingness to Communicate, English learning motivation, and self-confidence)	7	- Baran-Łucarz, 2014 - Chung and Leung, 2016 - Dewaele and Dewaele, 2018 - Liu, 2017 - Liu and Huang, 2011 - Tridinanti, 2018 - Zhou et al., 2020
	Relationship between FLA and sociolinguistic variables (e.g., gender, personal experience, language background, and immigrant status)	4	- Sevinc, 2018 - Sevinç and Dewaele, 2018 - Thompson and Lee, 2012 - Thompson and Lee, 2014
L2 speaking anxiety scales are adopted to unpack the relationship between anxiety and language performance variables.	Relationship between FLA and L2 speaking performance/L2 proficiency level	4	- Baran-Łucarz, 2011 - Baran-Łucarz, 2013 - Huang, 2018 - Zheng and Cheng, 2018

The next section of this paper features a narrative review of the scales that were initially used for assessing anxiety, followed by a documentation of L2 speaking anxiety scale development and validation process. The section, “*The application of L2 speaking anxiety assessment scales with a dynamic approach*,” explains the other themes identified from the articles at length and synthesizes the methods implemented to understand the role of anxiety in L2 learning.

## Scales for assessing L2 speaking anxiety

In comparison to more fine-grained frameworks that recognize anxiety of specific types, anxiety has been regarded as a manifestation of medical disorder, the assessment tools of which were designed from a pathological perspective. For example, Spitzer et al. (2006) documented the development process of Generalized Anxiety Disorders (GAD) scale, which consists of items evaluating the feeling of nervousness, losing control, over-worrying, and difficulty of relaxing through a 4-point Likert scale.

Scales assessing speaking anxiety have also witnessed a development trend that starts from measuring anxiety as a broader concept. The measurement of speaking anxiety, however, is closely connected with the Foreign Language Classroom Anxiety Scale (FLCAS) constructed by Horwitz et al. (1986), which served as the foundation for a plethora of different versions of speaking anxiety assessment scales. In addition, foreign language speaking has been represented by specific speech activities such as L2 pronunciation practices and L2 public speaking, which resulted in the compilation of more detailed scales. This section of paper enlists a historical review of the scales frequently used for assessing speaking anxiety, which would provide researchers with a wide range of options for investigating related research inquires.

### Test-based scales in evaluation of anxiety

Test-based scales measure anxiety as a situational concept, or more specifically, anxiety that occurs in a testing environment. Sarason (1984) defined anxiety as “a complex state that includes cognitive, emotional, behavioral, and bodily reactions” (p. 931), and specified the existence of test anxiety when the activities triggering anxiety take place in a context of academic evaluation. A large amount of efforts have been spent in developing Test Anxiety Scale (TAS) (Mandler and Sarason, 1952; Sarason, 1961, 1978, 1984). TAS consists of 39 true-or-false statements, which inquire respondents' cognitive, emotional, and behavioral reactions. Sample items include “If I know I was going to take an intelligence test, I would worry a great deal when taking it” and “Thoughts of doing poorly interfere with my performance on tests.” Anxiety, which is highly situational and individual, Is interpreted as a cognitive response characterized by one's feelings and doubts.

The difference between general anxiety and specific anxiety, or “the relative merits of situational specificity” has been mentioned in Alpert and Haber (1960, p. 208), which also explained the components of Achievement Anxiety Test (AAT). The 19-item AAT scale is composed of a 10-item Facilitating Anxiety Scale (FAS) and a 9-item Debilitating Anxiety Scale (DAS). Facilitating Anxiety Scale (FAS) foregrounds the positive connection between anxiety and productivity (e.g., “I work more efficiently under pressure, as when the task is very important.”), Debilitating Anxiety Scale (DAS), in contrast, includes statements disclosing the negative influence of anxiety on performance (e.g., “Nervousness while taking an exam or test hinders me from doing well.”). Respondents need to make a decision between “Always” and “Never” while answering the statements. In this study, specific anxiety scales have shown to be more efficient predictors for respondents' academic performance in comparison to general anxiety scales, which inspires the construction of scenario-based items in devising anxiety assessment scales.

Within the group of anxiety assessment scales, the Test Anxiety Inventory (TAI) (Spielberger, 1980) has been used on undergraduate students since the 1980s, the development of which is inseparable from the contribution of TAS. The TAI is a self-report scale consisting of 20 items with two subscales: (a) the “worry” subscale, which contains statements describing behavioral patterns in relation to test anxiety, such as “I believe I am going to fail the test.” (b) the “emotionality” subscale, which contains items stating physiological responses associated with test anxiety, such as “my heart beats faster when I am taking a test”. TAI adopts a 4-point Likert scale for evaluation, which ranges from “1 = almost never” to “4 = almost always”. High scores indicate more intensive anxiety perceived by respondents.

Based on the differentiation between state anxiety and trait anxiety, Spielberger et al. (1983) further categorized the State Trait Anxiety Inventory (STAI) as a 40-item self-report measurement tool. The scale also made use of a 4-point Likert scale ranging from “1 = not at all” to “4 = very much so”. Two 20-item subscales are included in the inventory: (a) the “state anxiety” subscale (STAI-S), representing the individual's anxiety level when he/she is answering the questionnaire. (b) the “trait anxiety” subscale (STAI-T), representing the respondent's overall anxiety level across a lengthy time span.

This collection of scales measuring anxiety, whether from a more general view or using detailed categorizing framework, laid the foundation of pinning down language learning as a situational specific activity. The anxiety assessment scales used for foreign language learning, or the research realm of Foreign Language Anxiety (FLA), will be introduced in the next section.

### Classroom-based scales in evaluation of speaking anxiety

To fulfill the purpose of assessing FLA, researchers have developed scales to measure learners' anxiety while using a specific language, or learning a foreign language in the classroom. Gardner and Smythe (1975) used the French Class Anxiety Scale as a predictor for students' intention to learn French. In addition, Gardner et al. (1979) adopted an 8-item instrument as the French Use Anxiety Scale to disentangle the relationship among learners' attitudes, motivation, as well as their language proficiency level. Similar scales were also applied to examine learners' anxiety for learning and testing in English (Clément et al., 1977, 1980) and Spanish (Muchnick and Wolfe, 1982).

From a broader perspective, foreign Language classroom anxiety, as was defined by Horwitz et al. (1986) as a “distinct complex of self-perceptions, beliefs, feelings and behaviors related to classroom language teaching arising from a uniqueness of the language learning process” (p. 128), is measured by Foreign Language Classroom Anxiety Scale (FLCAS) developed by Horwitz et al. (1986). As for FLCAS, three categories of performative anxieties were identified in relation to foreign language anxiety, i.e., communication apprehension, test anxiety, and fear of negative evaluation. FLCAS consists of 33 items with a 5-point Likert scale ranging from “Strongly Disagree” to “Strongly Agree”. Sample items include “In language class, I can get so nervous I forget things I know,” “I don't worry about making mistakes in language class,” and “I am usually at ease during tests in my language class”.

FLCAS has played a pivotal role in the adaptation and construction of scales related to L2 speaking anxiety. For example, Öztürk and Gürbüz (2013) designed the Foreign Language Speaking Anxiety Questionnaire, where the researchers selected 18 items from the 33 items of FLCAS. The 18 items were directly related to foreign language speaking anxiety. Also, Liu (2021) extracted 12 items from FLCAS, which are associated with learners' anxiety/confidence when speaking English. These items were grouped as the English Speaking Anxiety Scale (ESAS) in related research. FLCAS has also been translated into a variety of languages, including Hungarian (Tóth, 2007), Persian (Alidoost et al., 2013), Thai (Tanielian, 2014), and Arabic (Dewaele and Al-Saraj, 2015).

### Activity-based scales in evaluation of L2 speaking anxiety

Another group of speaking anxiety scales capture language learners' perception for specific speaking activities, which occur either in classrooms or during daily communication. Communication-bound anxieties have been extensively discussed in McCroskey (1970), where Personal Report for Communication Apprehension (PRCA) was developed to measure communication apprehension among individuals across different age groups. As for college students, or adult foreign language learners, the items of PRCA involve both interpersonal communication scenarios (e.g., making a conversation with an acquaintance) and small group communication (e.g., contributing to a small group discussion). A few items were also designed to evaluate communication apprehension in public speaking contexts. The PRCA questionnaire includes 20 items in total, and respondents were asked to use a 5-point Likert scale for assessment purposes.

A dual conceptualization of L2 speaking anxiety was also advocated by Woodrow (2006), as speaking activities happen both within classrooms for pedagogical purposes and in daily life to fulfill communicative goals. Woodrow (2006) mentioned that speaking anxiety “has a debilitating effect on the performance of speakers of English as a second language” (p. 308). The Second Language Speaking Anxiety Scale (SLSAS) constructed in Woodrow (2006) thus adopts the classification scheme of “in-class anxiety” and “out-of-class anxiety”. SLSAS is composed of 11 items of in-class anxiety, 11 items of out-of-class anxiety, and 5 yes/no statements in description of the respondent's general personality. Contexts related to in-class activities include giving presentations and contributing to formal discussions, while stressors of out-of-class activities involve asking/answering questions and starting conversations with L1 English speakers.

In addition to incorporating daily communication scenarios, speaking anxiety scales are also represented by activities of more concrete forms. For example, Public Speaking Anxiety (PSA) has been recognized as a situation specific FLA overlapping with social anxiety. Efficient public speaking, as claimed by Lucas (2013), embodies “critical thinking, creative ideas, and logical construction” and has become a prominent teaching component on the syllabus of college English oral communication courses. Personal Report of Public Speaking Anxiety (PRPSA) adapted from McCroskey (1970) was used by Zheng et al. (2021) to evaluate anxiety in connection to English public speaking. PRFSA consists of 34 items with a 5-Likert scale. Sample items are constructed based on public speaking scenarios, such as “Although I am nervous just before starting an English public speech, I soon settle down after starting and feel calm and comfortable” and “While giving an English public speech, I get so nervous I forget facts I really know.”

Public Speaking Anxiety (PSA) has also been measured as one category of social anxiety through the lens of psychometric studies, where L1 speakers are recruited as participants. For instance, the Public Speaking Anxiety Scale (PSAS) introduced by Bartholomay and Houlihan (2016) measures cognitive, behavioral, and psychological anxiety based on the 3-component anxiety model proposed in Lang (1971). The 17-item self-report assessment tool uses a five-point Likert scale to measure public speaking anxiety. Another set of scale is named as Personal Report of Confidence as Speaker (PRCS), or a 12 true or false items adapted from Gilkinson (1942).

Apart from the scales designed in assessment of Public Speaking Anxiety, pronunciation practices in language learning classrooms are also the targets for L2 speaking anxiety evaluation. Baran-Łucarz (2013) developed Phonetics Learning Anxiety Scale (PhLA) to assess the level of anxiety language learners experience during a phonetics course. Pronunciation anxiety was clarified as a measurable dimension, which could be analyzed through self-perception of pronunciation, fear of negative evaluation, and beliefs concerning the pronunciation of the target language. The PhLA scale is a 44-item self-report questionnaire, in which a 6-point Likert scale is applied. The first part of PhLA includes 15 items that measure the general phonetics learning anxiety level, such as students' attitudes toward the phonetics class and identification of the cognitive symptoms of anxiety (e.g., “I am so nervous that I can't hear the new sounds of word stress properly”). The second section of PhLA contains 20 items, which aim at assessing L2 learners' concern of mistakes, oral performance apprehension, pronunciation self-image, pronunciation self-assessment, as well as test anxiety and learners' beliefs of pronunciation learning. Sample items for the second section include “I feel more embarrassed committing a pronunciation mistake than any other type of mistake” and “I think I sound ridiculous pronouncing English sounds and words the way they should be pronounced”.

## Methods of examining the reliability and validity of L2 speaking anxiety scales

As a prerequisite for applying assessment tools in a reliable and efficient manner, examining the reliability and validity of a scale is a necessary step for researchers to accomplish before reporting data analysis results. Speaking anxiety scales have been investigated through both Classical Testing Theory (CTT) approaches and probabilistic methods, the latter of which also formed an individual research strand in scale development and interpretation.

From the perspective of Classical Testing Theory (CTT), statistics in support of scale reliability and validity include Cronbach's alpha, test-retest reliability, and the correlational results between the scale to be examined and other established assessment instruments. Other frequently-used statistical methods include Exploratory Factor Analysis and Confirmatory Factor Analysis, which are capable of extracting the dimensions represented by multiple items on the scale. For example, Mak (2011) conducted Factor Analysis on FLCAS results collected from Chinese L2 English speakers, which revealed five factors in relation to students' in-class speaking anxiety. These five factors are explained as: “speech anxiety and fear of negative evaluation,” “uncomfortableness when speaking with native speakers,” “negative attitudes toward the English classroom,” “negative self-evaluation,” and “fear of failing the class/consequences of personal failure”.

Factor Analysis has also been used to examine newly-developed scales. Yaikhong and Usaha (2012) constructed the Public Speaking Class Anxiety Scale (PSCAS), the Items on which were drawn from existing scales that assess L2 speaking anxiety. The researchers calculated Cronbach's alpha coefficient to test the internal consistency of PSCAS, and also used Factor Analysis to identify a list of components the new instrument contains. These statistics in combination have provided supporting evidence for the construct validity of the newly-developed scale. Similar methods have been used to analyze the structures of speaking anxiety assessment scales adopted in diverse L1 contexts (Park, 2014; Ali, 2016, 2017; Taat et al., 2020). The purpose of conducting Factor Analysis, however, is not restricted to examining the questionnaire's validity and reliability. As the dimensions presented by Factor Analysis vary across L2 English learners with different L1 backgrounds and in age groups, the results have also helped researchers pinpoint the sources of anxiety more accurately and explored for pedagogical implications accordingly.

In parallel with statistical methods grounded on the Classical Testing Theory (CTT), Rasch measurement has also been adopted for scale interpretation as a probabilistic method. Item analysis conducted within the CTT framework relies on the assumption that the Likert scales used by individual participants are interval in nature, where the distance between “1 = Strongly Disagree” and “2 = Disagree” is equal to that between “3 = Agree” and “4 = Strongly Agree”. Rasch measurement, however, transforms the Likert scales to logit scales. Both item difficulties and human factors are thus put into consideration for result interpretation.

Multiple scales measuring learner anxiety have been analyzed through Rasch modeling. For example, Apple (2013) conducted Rasch analysis on FLCAS, which was used among Japanese college students. According to Apple (2013, p. 21):

“Researchers can use Rasch analysis to take into account measurement error, item location, person location, and fit statistics to better determine the degree to which speaking anxiety levels exist for individual students as well as to determine the degree to which speaking anxiety level exists for individual students as well as to determine which questionnaire items were the best indicators of speaking anxiety.”

More recent studies featuring Rasch analysis of speaking anxiety scales include Lin et al. (2021), which examined the psychometric properties of the self-reported Public Speaking Anxiety Scale (PSAS) introduced by Bartholomay and Houlihan (2016). Lin et al. (2021) reported that although no systematic bias was detected in responses for age or gender, the PSAS demonstrated evidence of multidimensionality. The issue was resolved after splitting the scale into two discrete subscales: Emotional and Physiological. When scales are used among individuals with diverse backgrounds in L1, home culture, or language proficiency level, Rasch analysis could help explain the functioning of scales with sufficient details. The necessity of dividing questionnaires into different sections or subscales is often brought into attention, which provides researchers with abundant opportunities to re-interpret anxiety, the subconstructs of anxiety, and the interrelationship among multiple dimensions that surfaced from the same scale.

## The application of L2 speaking anxiety assessment scales with a dynamic approach

Scales measuring L2 speaking anxiety have been used for multiple purposes in studies related to language learning. A few themes could be identified from the state-of-art research listed in Table 1, which range from assessing pedagogical outcome to explaining the relationship between speaking anxiety and variables concerning language performance. The evolvement of the dynamic approach to understanding anxiety has also led to novel explanations of its effects on foreign language learning. This section of paper maps out a research outline regarding L2 speaking anxiety, which has been quantifies by different sets of scales. The interpretation of the dynamic approach is also discussed, which hopefully would offer new insights into linking anxiety assessment results with pedagogical support provided in language classes.

### Theme 1: Speaking anxiety scales in assessment of pedagogical outcome

An important usage of speech anxiety scales is to examine the effects of a myriad of pedagogical designs in language teaching classrooms, which often bear the purpose of reducing FLA. The instructional methods implemented in language classrooms, however, are usually housed within a certain pedagogical framework. For example, Lee (2016) examined the effectiveness of oral corrective feedback on international graduate students' speaking anxiety. The categorization of corrective feedback forms the backbone of the study, where learners received different formats of feedback from their instructors. Anxiety is considered as an affective variable, with scales assessing learners' anxiety level being the major research instrument.

EFL speaking classes also witnessed the application of the pedagogical approach that experiments with establishing a community of practice. In Kralova et al. (2017), the researchers designed a psycho-socio training program to reduce the foreign language pronunciation anxiety of L2 English pre-service teachers. The psycho-socio training program is composed of interventional sessions that help pre-service teachers cope with anxiety by understanding their own pronunciation through other group members' emotions and behaviors. The Foreign Language Pronunciation Anxiety (FLPA) scale, which was adapted from both the Foreign Language Classroom Anxiety Scale (Horwitz et al., 1986) and the Phonetics Learning Anxiety Scale (Baran-Łucarz, 2013), was used for evaluating participants' English pronunciation anxiety level before and after the intervention. FLPA includes 20 declarative statements to probe into learners' perceptions of their pronunciation, during which the participants were asked to use a 6-point Likert scale to indicate the extent to which they agree/disagree.

When situated in a classroom learning environment, anxiety has been researched through a variety of assessment approaches. In Zheng et al. (2021), self-assessment and peer-assessment were arranged in different sequences before L2 learners completed delivering public speeches in English. The Personal Report of Public Speaking Anxiety (PRPSA) adapted from McCroskey (1970) was used to monitor the change of anxiety level among students, indicating that formative practices with self-assessment implemented first have efficiently reduced the impact of speaking anxiety. Dynamic assessment, which is characterized by scaffolded feedback and full recognition of learners' potentials, is also becoming a widely-accepted assessment approach in identifying the change of anxiety level among L2 learners. Chen et al. (2022) illustrated that speech recognition system has alleviated L2 learners' speaking anxiety to a larger extent when used with the guidance of dynamic assessment. Also, dynamic assessment has been used to build a more socially constructive classroom environment for EFL learners (Sohrabi and Ahmadi Safa, 2020). Estaji and Farahanynia (2019), on the other hand, discussed the effectiveness of more nuanced dynamic assessment approaches on L2 learners' speaking anxiety, and explicated the differences between interactive and interventional dynamic assessment.

Another line of speaking anxiety research is stimulated by a stronger presence of technology in classrooms and the global impact of the COVID-19 pandemic. As Aydin (2018) advocated, technology is not the only factor that is inducing debilitating or facilitating anxiety over language learning. A dynamic approach is thus needed to comprehend the interrelationship among technology, anxiety, and other language learning variables. Multiple scales have thus been applied to quantify FLA caused by different reasons. In a virtual classroom equipped with instructional technology support, Xiangming et al. (2020) investigated the technological affordances of foreign language learners through observing their language performance scores as well as anxiety level fluctuation. In this study, the possible influence caused by technology was examined by FLCAS in combination with Self-Recalled Anxiety Changes (SRAC). As a complementary assessment instrument to FLCAS, SRAC is a 7-point Likert scale that records student evaluation of one single item at multiple time spots during a 16-week semester: “Please recall and record your learning anxiety level in week 1 (or week 4 or week 7 or week 10)”. In a technology assisted learning environment, the concerted use of FLCAS and scales related to learning behaviors has presented language teachers with informative results to devise strategies for handling anxiety.

In terms of instructional technologies, Chen and Hwang (2020) researched the influence of flipped learning on EFL learners' speaking anxiety. In the flipping classroom mode, students navigated through the learning materials at their own pace, and adopted concept mapping as a strategy to organize their thoughts and ideas for classroom discussion. The Second Language Speaking Anxiety Scale (SLSAS) developed by Woodrow (2006) was used to assess EFL learners' speaking anxiety after using the mapping approach. Students' ratings for the anxiety were correlated with the measurement results for critical thinking awareness as well. Studies inspecting the effects of instructional technology also include Bárkányi (2021) and Pan et al. (2022), in which the influence of Massive Open Online Course (MOOC) and virtual interaction on EFL learners' foreign language speaking anxiety forms the major question.

The involvement of technology in speaking pedagogy is also manifested by the application of web-based software in classrooms. Bashori et al. (2022) tested whether web-based language learning might alleviate speaking anxiety, and invited L2 English speakers to participate in two Automatic Speech Recognition (ASR) experiments. L2 English learners responded to both Foreign Language Classroom Anxiety Speaking (FLCAS) (Horwitz et al., 1986) and Foreign Language Speaking Anxiety Scale (FLSAS) (Öztürk and Gürbüz, 2013). The level of FLSAS is higher than FLCAS, which corroborated with an assumption that speaking is the most anxiety provoking activity. However, students' anxiety level did not experience a significant drop after using the ASR application, implying that successful in-class implementation of web-based learning technology might need a larger amount of instructor guidance and technological support. The challenges encountered by L2 English learners through online communication have been discussed in a number of studies focusing on instructional technology, where Computer-Mediated Communication (CMC) is realized in virtual classrooms through technological advancement at full speed (e.g., video, chat, voice, and virtual technology) (Satar and Özdener, 2008; Jebali, 2014; York et al., 2021; Aldukhayel, 2022).

### Theme 2: Speaking anxiety scales in connection with affective variables: A dynamic approach

As was mentioned in MacIntyre (2017), the interpretation of the connections between language learning anxiety and affective variables, such as attitudes, motivation, and Willingness to Communicate (WTC), is undergoing a shift toward the dynamic approach. The relationship between anxiety and affective variable has been explored in Baran-Łucarz (2014), in which learners' pronunciation anxiety was quantified and correlated with the measurement results for WTC. The study suggested that higher pronunciation anxiety would lead to lower WTC, and this pattern looms to be the most obvious for L2 speakers at intermediate anxiety level. In Liu (2017), L2 Chinese college students' speaking anxiety was also found to be negatively correlated with WTC. The negative impact of speaking anxiety is urging language teachers to scaffold language learning tasks and help L2 students familiarize with the target language culture, which might curb the effects of anxiety on using L2 for communicative purposes.

The dynamic approach to understanding anxiety, which is also represented by the interplay of variables such as age, L1 background, gender, and L2 proficiency level, has witnessed a growing body of “moderator” research in explanation of the negative impact caused by anxiety. Thompson and Lee (2014)'s study found that language learners' experience abroad and L2 proficiency were jointly related to their ratings of anxiety. Both Sevinc (2018) and Sevinç and Dewaele (2018) delved into the possible impact of immigration status and language background on heritage language speaking anxiety. In addition, Chou (2018) examined the influence of full and partial English Medium Instruction on L2 learner's anxiety level. Students receiving partial English Medium Instruction exhibited higher level of speaking anxiety and a lack of confidence. The pool of “moderator” factors is still expanding, which is enriched by language learners' individual background information and personal learning experience.

The dynamic interactions among all the factors are also symbolized by new perceptions of the relationship between anxiety and joy of language learning, or a re-conceptualization of facilitating anxiety and debilitating anxiety. In contrast with FLCAS, Dewaele and MacIntyre (2014) developed the scale of Foreign Language Enjoyment (FLE), which includes statements inquiring learners' attitudes toward a foreign language, the atmosphere in classroom, and the friendliness of language teachers. Results extracted from scales such as FLCAS and FLE are representative of the underlying constructs they measure, and the relationship between the scales reflects the dynamic interaction among different dimensions involved. From a pedagogical perspective, however, high level of enjoyment, does not necessarily lead to a low level of anxiety. A “constructive balance” (p. 262) needs to be maintained despite of the fact that successful learners would report rating scores slightly higher in enjoyment.

To search for interpretations of the non-linear relationship between anxiety and joy, Dewaele and MacIntyre (2014) discussed the theory of positivity ratio by resorting to Fredrickson (2013), who has suggested that the ratio of positive to negative emotions might be more prominent than the absence of negative emotion for predicting or evaluating L2 learners' performance. In Dewaele and MacIntyre (2014), the ratio of positive to negative emotion in the most advanced group of learners is “approximately 2:1, then 11/2:1 in the intermediate group, and finally 1:1 in the group self-described as performing far below average.” The discussion was continued in Dewaele and Alfawzan (2018), as correlation results show that the positive effects of FLE on L2 learners' performance outweigh the negative effects of FLCAS on L2 learners' test performance. Furthermore, Chen et al. (2021) examined the interactions of trait emotional intelligence (trait EI), foreign language anxiety (FLA), and foreign language enjoyment (FLE) in the foreign language speaking classroom, where trait EI was found to be significantly interacting with both FLA and FLE. Investigation of the connections between enjoyment and anxiety, as well as the involvement of other affective factors, reflects the influence of dynamic approach on reconstructing the role of anxiety in language learning. The consideration of individual factors, such as studying abroad experiences, generational differences, and immigration status would also largely benefit the understanding of L2 speaking anxiety, as learners' reporting of anxiety level may differ across diverse backgrounds and everchanging contexts.

### Theme 3: Speaking anxiety scales in connection with language performance factors: Statistical modeling

In search of explanations for the interactions among speaking anxiety and language performance factors, researchers have also harnessed the explanatory power of statistics in related studies. This trend frequently occurs when researchers are interpreting the relationship between speaking anxiety and learners' language performance. Variables that are predictive of language performance, such as students' language proficiency level, their perceived language competence level, and language test scores, are also added to research questions together with affective variables. As explicit indicators of students' learning achievement, both students' academic performance and language performance have been used as variables displaying the influence of speaking anxiety. Botes et al. (2020) conducted a meta-analysis on the connection between FLCAS (Horwitz et al., 1986) and language learners' academic achievement (i.e., general academic achievement, reading, writing, speaking, and listening academic achievement). Results showed that FLCAS has a moderate correlation with speaking academic achievement, and the majority of studies indicated a negative correlation between FLCAS and speaking achievement. Dikmen's (2021) meta-analysis presented similar findings in terms of the negative impact of FLA on learners' performance, but also pointed out the “moderator” effect of types of anxiety. According to Dikmen (2021), listening anxiety decreased students' EFL performance the most.

In addition to meta-analysis, correlational analysis is one of the most straightforward statistical procedures for analyzing the connection between anxiety and language performance variables. Baran-Łucarz (2011), for example, examined L2 English learners' performance on a pronunciation test, along with the learners' self-assessment measures for pronunciation and FLCAS. Results showed that perceived pronunciation level is more strongly correlated with anxiety, which articulated the necessity of designing efficient self-assessment tasks in pronunciation courses for anxiety reduction. A supportive classroom with positive dynamics would be beneficial to controlling students' fear of making errors.

Statistical methods applied for analyzing anxiety also include Structural Equation Modeling (SEM), the application of which is based on the abundant scale measurement results yielded by anxiety research. Chung and Leung (2016) inspected the structural relationship among English language learning motivation, foreign language speaking anxiety, perceived English competence, willingness to communicate, English learning engagement, and motivational intensity among L2 English speakers in Hong Kong. Anxiety is measured through the Public Speaking Class Anxiety Scale (McCroskey, 1970; Horwitz et al., 1986; Yaikhong and Usaha, 2012). Two significant SEM models are established, with Model 1 recognizing that both integrative and instrumental motivation are significant predictors of speaking anxiety. Speaking anxiety is also a significant predictor of WTC. Model 2, however, illustrates that WTC could also be significantly predicted by perceived English competence.

Path analytical modeling was also used in Huang (2018), who collected the measurement results for four anxiety assessment scales and the speaking score achieved by L2 learners at a large-scale standardized test. The study also aims at exploring the interactions occurring between all the scales and students' speaking test scores. The four assessment scales included in this study are: The Trait Anxiety Inventory, the State Anxiety Inventory, the English Classroom Anxiety Scale adapted from FLCAS, and the Text Anxiety Scale. Statistical analysis showed L2 learners' speaking test performance is significantly impacted by trait anxiety and language anxiety. Both trait anxiety and language anxiety are direct sources for state anxiety, the latter of which is indirectly attributed to test anxiety.

The broad selection of statistical methods in anxiety-related research has carved out a space for chances of data-mining. Unleashing the potential of statistical analysis, however, cannot be separated from an evolving understanding of anxiety as a measurable construct and an appropriate use of assessment scales. The development, validation, and application of assessment instruments is thus of great importance for future research, which will be further discussed in the conclusion section of this paper.

## Concluding remarks

This paper presents a historical review of the scales commonly used to assess L2 speaking anxiety, and discussed the statistical methods applied for assuring the reliability and validity of scales. The dynamic approach to understanding anxiety, which has been reflected in recent studies published, is providing researchers with more diversified directions in configuring the relationship among anxiety, affective factors, and variables related to language learners' performance. In addition, the themes identified from anxiety-related research render some new thoughts about some future research questions:

*(1) The impact of technology on language learners' speaking anxiety*

While omnipresent technology is altering the landscape of language instruction, the complication caused by COVID-19 pandemic has led to a series of “unwanted” situations, such as limited opportunities for face-to-face contact, oral English communication courses and tests “accidentally” transformed into an online format, as well as job interviews in English that are conducted through a chatting room in cyber space. Learners of English as a second language are thus coping with challenges both interpersonally and technologically, which might become the new norms for their future academic/professional career. The scales of assessing L2 speaking anxiety, in this case, could be used in tandem with questionnaires evaluating social anxiety or technology anxiety to achieve a well-rounded understanding of all the stressors. Technology-related/testing related scales include the Attitude Toward Computerized Testing Scale (ATCAS) developed by Smith and Caputi (2004), in which respondents' cognitive and affective reactions toward computerized tests were also assessed.

*(2) The impact of pedagogical interventions on language learners' speaking anxiety*

From the perspective of L1 speaking research, speaking anxiety is sometimes treated as a speech disorder. Technology interventions such as Virtual Reality (VR) have been applied to reduce anxiety through exposure therapy (Lindner et al., 2021; Reeves et al., 2021), where speakers are placed in scenarios inducing anxiety and become strategically prepared for authentic communication. After Virtual Technology exposure therapy (VRET) sessions, patients needed to finish speaking tasks in contexts that stimulate daily conversation environment. It is highly problematic to mix L2 speakers, who are experiencing challenges of learning a new language, with L1 patients diagnosed with speech orders. The therapy sessions that are tentatively exploring for possible stressing scenarios, however, might lend new ideas to the design of L2 oral communication classes. For most of the times, the renovation of pedagogical approaches and instructional design has successfully reduced language learners' anxiety level. Creating activities that integrate the theory of “positivity ratio” would probably reveal the positive side of anxiety, which might be beneficial to students' language performance.

## Limitations of the study

This review attempts to retrace the instruments that have largely contributed to the content development of scales assessing L2 speaking anxiety. Scales such as Test Anxiety Scale (TAS), Generalized Anxiety Disorders (GAD) scale, Test Anxiety Inventory (TAI), State Trait Anxiety Inventory (STAI), and Foreign Language Classroom Anxiety Scale (FLCAS) have been described at length as highly relevant to the construction of scales assessing L2 speaking anxiety. It is still possible, however, that the development of new scales has resorted to instruments that are beyond the literature surveyed in this paper.

It also needs to be pointed out that the assessment of L2 speaking anxiety is often embedded in the measurement of FLA in general. The interpretation of speaking anxiety, in this case, is in close connection with other types of anxiety (e.g., test anxiety, listening anxiety, writing anxiety). Within the 49 peer-reviewed articles selected by the author, L2 speaking is a highlighted activity investigated by the researchers. However, this review report would benefit from examining scale development literature regarding other language skills. Extending the scope of reviewed articles can help provide more insightful suggestions for compiling speaking anxiety assessment scales, which will better accommodate various research needs and multiple language learning contexts.

## Data availability statement

The original contributions presented in the study are included in the article/supplementary material, further inquiries can be directed to the corresponding author.

## Author contributions

The author confirms being the sole contributor of this work and has approved it for publication.

## Funding

This paper represents independent research funded by the Chenguang Scholar Grant (Grant Number 20CG04), which is jointly provided by the Shanghai Municipal Education Committee and Shanghai Education Development Foundation.

## Conflict of interest

The author declares that the research was conducted in the absence of any commercial or financial relationships that could be construed as a potential conflict of interest.

## Publisher's note

All claims expressed in this article are solely those of the authors and do not necessarily represent those of their affiliated organizations, or those of the publisher, the editors and the reviewers. Any product that may be evaluated in this article, or claim that may be made by its manufacturer, is not guaranteed or endorsed by the publisher.
